# General-Purpose Genotype or How Epigenetics Extend the Flexibility of a Genotype

**DOI:** 10.1155/2012/317175

**Published:** 2011-12-15

**Authors:** Rachel Massicotte, Bernard Angers

**Affiliations:** ^1^Département de Sciences Biologiques, Université de Montréal, C.P. 6128, succursale Centre-ville, Montréal, QC, Canada H3C 3J7; ^2^Group for Interuniversity Research in Limnology and Aquatic Environment (GRIL), Trois-Rivières, Qc, Canada G9A 5H7

## Abstract

This project aims at investigating the link between individual epigenetic variability (not related to genetic variability) and the variation of natural environmental conditions. We studied DNA methylation polymorphisms of individuals belonging to a single genetic lineage of the clonal diploid fish *Chrosomus eos-neogaeus* sampled in seven geographically distant lakes. In spite of a low number of informative fragments obtained from an MSAP analysis, individuals of a given lake are epigenetically similar, and methylation profiles allow the clustering of individuals in two distinct groups of populations among lakes. More importantly, we observed a significant pH variation that is consistent with the two epigenetic groups. It thus seems that the genotype studied has the potential to respond differentially via epigenetic modifications under variable environmental conditions, making epigenetic processes a relevant molecular mechanism contributing to phenotypic plasticity over variable environments in accordance with the GPG model.

## 1. Introduction

Over the years, the debate about the evolutionary advantage of sexual over asexual reproduction has focused in part on the higher adaptive potential of populations with standing genetic variation [1] (and references therein). Each generation, the reproduction of amphimictic organisms results in genetic mixing, thus creating a multitude of new genotypes (and potentially novel phenotypes) in natural populations. While in sexually reproducing organisms each individual possesses a different genotype, asexually reproducing individuals from the same clonal lineage are presumed to be genetically identical. 

**Figure 1:**
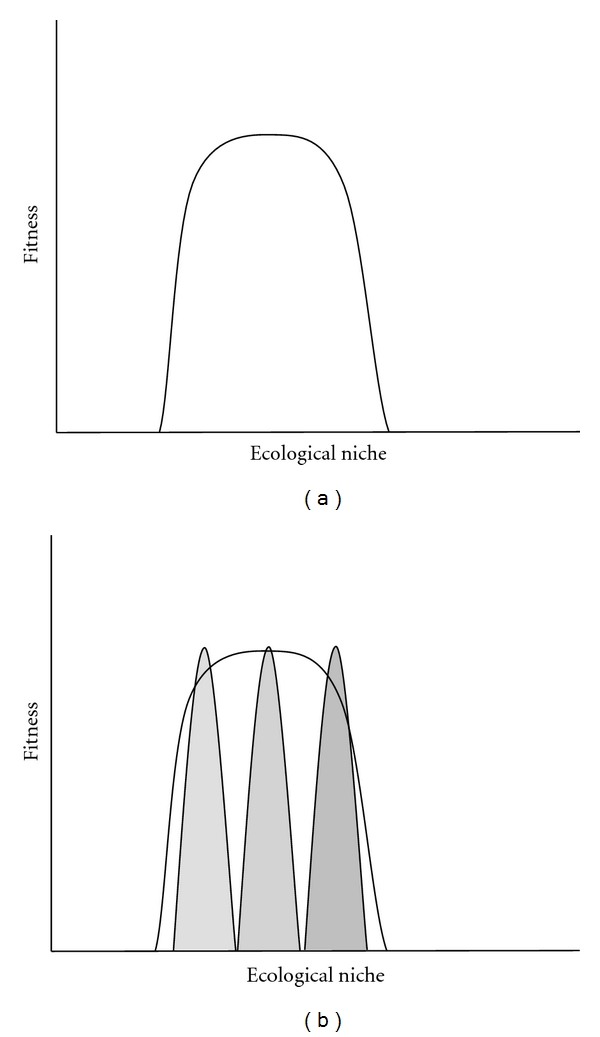
Graphic representation of the general-purpose genotype (GPG) model and the flexibility hypothesis. (a) GPG model, a flexible genetic lineage (unfilled distribution) with a wide ecological niche and a high fitness under variable environmental conditions. (b) Epigenetic as a mechanism extending the flexibility of a genome, environmentally induced epigenotypes (grey distributions) from a single genetic lineage (unfilled distribution from (a)).

On the other hand, asexuality has some advantages of its own; there is no need to produce males, and asexual populations can double their size each generation [2]. This twofold advantage of asexual reproduction is thought to be constrained by their limitation in colonizing new environments and/or when living in temporally unstable or heterogeneous environments. In such conditions, the survival, flexibility, and adaptive potential of asexual lineages are aspects that are not well understood. The general-purpose genotype (GPG) model [3] (Figure 1(a)) proposed that evolutionary success of asexual organisms could be possible via generalist lineages selected for their flexible phenotypes utilizing wide ecological niches. Such phenotypic flexibility enables a given genotype to be successful in many different and variable environments [4, 5]. Other models, such as the frozen niche variation (FNV) model [6], rely on the existence of genetic diversity among multiple highly specialized clonal lineages within a population each having respective narrow ecological subniches to explain the maintenance of asexual lineages. Each specialist lineage persists through time by partitioning of available ecological space so as to avoid clonal competition. However, microniche models do not provide explanations for how single clonal lineages can be successful across different and temporally variable environmental conditions.

One of the process underlying the GPG model is the concept of phenotypic plasticity, an environmentally induced phenotypic difference that occurs within an organism's life-time in the absence of genetic variation [7] (but see [8]). Epigenetic variation potentially represents a molecular mechanism that can generate phenotypic plasticity under natural environmental conditions [9]. The modification of the epigenome of an organism by variable methylation of DNA sequences has been shown to play a role in the regulation of some genes expression [10]. There are now numerous examples of epigenetically driven phenotypic variations that are not related to DNA sequence encoded genetic polymorphisms [11–14]. Such phenotypic variation can also be caused by an inability to maintain the original epigenetic state during embryogenesis [15]. Environmental cues (extrinsic signal) such as the diet [11, 16], temperature [17], maternal behaviour [14] and chemicals exposure [18], have been shown to influence the epigenetic profile of individuals. 

The fact that the genome is able to integrate extrinsic signals from the environment to vary gene expression is a potentially important mechanism for producing phenotypic plasticity. This stands in sharp contrast with better understood mechanisms which are based on sequence encoded genetic variation. More importantly, some epigenetic variation has been shown not to be related to genetic polymorphism in natural populations [19]. While the genome provides the material to work upon, it is the epigenetic regulation that in part enables genomic flexibility. Finally, recent studies have argued that some naturally occurring epimutations can be adaptive [11, 20].

This project aims at investigating the link between individual epigenetic variability (not related to genetic variability) and the variation of natural environmental conditions. In accordance with the general-purpose genotype (GPG) model, a flexible genotype under different environmental conditions would exhibit distinct methylation patterns due to alternate gene expression profiles necessary to produce flexible phenotypes (Figure 1(b)). As a result, DNA methylation would represent a molecular mechanism extending the plasticity and flexibility of phenotypes produced by a given genotype. As a model, we used the clonal fish hybrid *Chrosomus eos-neogaeus *(Cyprinidea and Pisces). We chose this system because a given clonal lineage of *C. eos-neogaeus *can be present over a large geographic distribution [21], is found in many different types of habitats [22], is thought to be generalist [23, 24], and, more importantly, has been shown to be epigenetically variable [19].

## 2. Materials and Methods

### 2.1. Biological Model and Sampling

The all-female *C. eos-neogaeus* taxon resulted from hybridization events between female finescale dace (*C. neogaeus*) and male northern redbelly dace (*C. eos*) [25]. The diploid hybrids reproduce clonally via gynogenesis [26, 27]. Sperm from one of the parental species is thus required but only to trigger embryogenesis: the resulting offspring are generally genetically identical to the mother [26]. In this complex, the paternal genome can be incorporated into the zygote [22, 26, 28] resulting in triploid or mosaic hybrids which differ in the proportion of diploid-triploid cell lineages [25].

**Figure 2:**
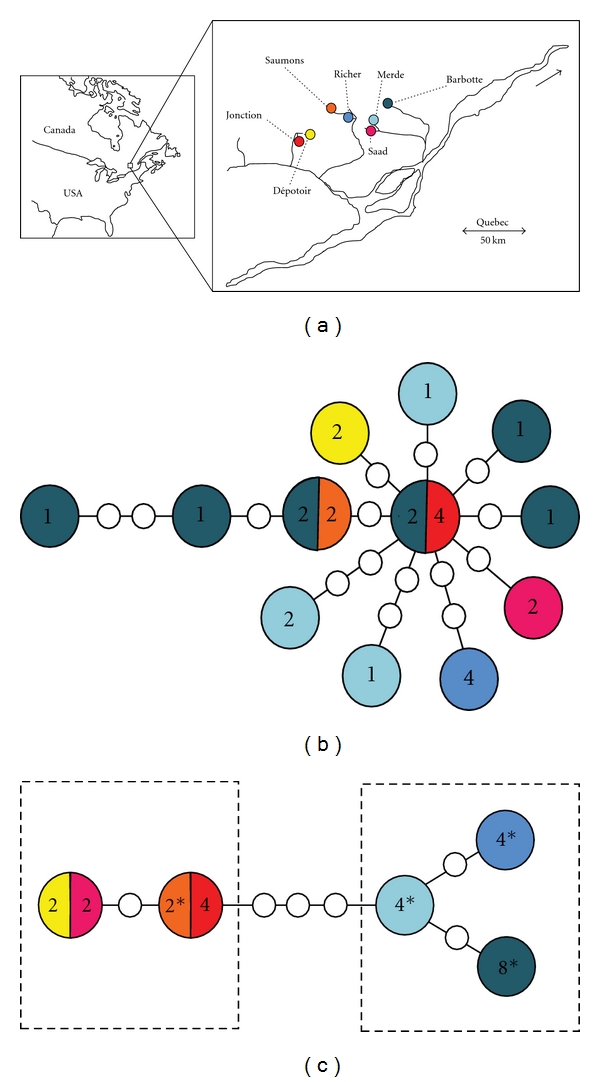
Details of sampling, genotypes, and epigenotypes diversity. (a) Sampled lakes in the Laurentian region, QC, Canada. (b) Minimum spanning network of the 12 genotypes identified by scoring nine microsatellite loci. The number of gynogenetic hybrids of each genotype per lake is indicated. (c) Minimum spanning network of the five main epigenotypes and two epigenetic groups (dash boxes) identified by the MSAP analysis. The number of gynogens of each epigenotype per lake is indicated. *refers to intrapopulation variation. The colour code of the sampled lakes from panel (a) is maintained throughout the rest of the figure.

Fish from seven lakes belonging to different watersheds of the St. Lawrence River, QC, Canada (Table 1; Figure 2(a)) were sampled in the reproduction season and over a short period of approximately two weeks. Total DNA from muscle tissue of parental species, three *C. eos* and three *C. neogaeus*, and 26 gynogenetic hybrids belonging to seven different lakes were extracted by proteinase K digestion followed by phenol-chloroform purification and ethanol precipitation [30]. The lakes sampled were each classified as one of the four different types of environment according to a characterization previously used to describe *C. eos-neogaeus* populations [29], water pH, and temperature were also measured. Total body length, total body weight, and gonads weight were measured for each individual in order to estimate the gonadosomatic index (GSI) and the Fulton's K condition factor index (K) [31]. The lakes sampled are known to contain either one or both parental species (*C. eos* and *C. neogaeus*) as well as gynogenetic and triploids hybrids [21, 28].

### 2.2. Genetic Identification

The gynogenetic hybrids were identified according to Binet and Angers [28]. Briefly, *C. eos-neogaeus* hybrids were identified using diagnostic markers designed on two genes. Primers of each marker were designed to provide PCR products of different sizes for *C. eos* and *C. neogaeus*, allowing chromosome identification. Individuals that displayed alleles of both parental species were classified as gynogenetic hybrids.

Gynogenetic hybrids (diploid) were then discriminated from triploid hybrids according to the ploidy level of the nuclear genome by using nine hypervariable microsatellites as detailed in Binet and Angers [28] and Angers and Schlosser [21]. Gynogens are expected to be hemizygous at every species-specific locus, while triploid hybrids (*C. eos*-*neogaeus* x* eos*) are expected to be heterozygous at loci specific for *C. eos* species. The microsatellites analysis also enabled the identification of the clonal lineage [21] and the discrimination of derived mutations. Only gynogenetic hybrids (diploid) were used for further analysis.

### 2.3. MSAP Analysis

We investigated epigenetic polymorphism at CCGG motif via an MSAP analysis [32] performed on parental species, three *C. eos* and three *C. neogaeus*, and the 26 *C. eos-neogaeus* gynogenetic hybrids identified in the procedure mentioned above. Each DNA sample was, respectively, digested with MseI/HpaII and MseI/MspI to allow the detection of differentially methylated sequences. Aliquots (4 *μ*L) of each sample for each primer combinations were loaded on 6% polyacrylamide gels (19 : 1 acrylamide to bisacrylamide) containing 8 M urea and 1X TBE. Fragments that displayed methylation polymorphism among samples at restriction sites were identified by the presence/absence banding pattern between the two treatments. Full methylation of both cytosines and hemimethylation of the internal cytosines cannot be investigated by MSAP. As a consequence, it was impossible to distinguish these fragments from unmethylated sequences. 

## 3. Results

### 3.1. Genetic Polymorphism: Microsatellite Loci Analysis

The analysis of nine highly variable microsatellite loci indicates that all samples belong to the same clonal lineage (lineage B6, [21]). Survey of microsatellite variation detected 14 mutations over nine loci and twelve multilocus mutant genotypes were identified within the clonal lineage (Figure 2(b)). These genotypes display very little divergence, since all but one genotype differ by only one or two mutations from the putative ancestral clone, with an average of 2.3 mutations among genotypes. The number of sublineages carrying derived mutations per lake varied from one to six (Table 1).

### 3.2. Epigenetic Polymorphism: MSAP Analysis

A total of 257 reproducible fragments detected between 150 and 600 bp were assessed with a set of six primer pairs. Over the 257 fragments detected in *C. eos-neogaeus* hybrids, 60 were exclusive to *C. neogaeus*, 67 to *C. eos*, and 114 were present in both parental species genomes. The remaining 16 fragments detected could not be associated to either of the parental species genomes. Eight fragments (3.11%) revealed informative methylation polymorphism among populations. Three fragments exclusive to *C. eos*, three fragments exclusive to *C. neogaeus*, and two fragments that were present in both parental species genome were differently methylated for some *C. eos-neogaeus* hybrids. The number of epigenotypes per lake varied from one to four (Table 1) and is not correlated with the number of samples (*R* ^2^ = 0.07, *P* = 0.56).

Two of the eight fragments are variable within populations, while the others are only variable among populations. For the 6 fragments that varied among populations, five main epigenotypes were detected. Although the sample size is low for some populations, individuals from a given population consistently shared the same methylation profile (Figure 2(c)). In most instances, individuals could be regrouped according to the lake of origin on the basis of their unique methylation profile.

**Figure 3:**
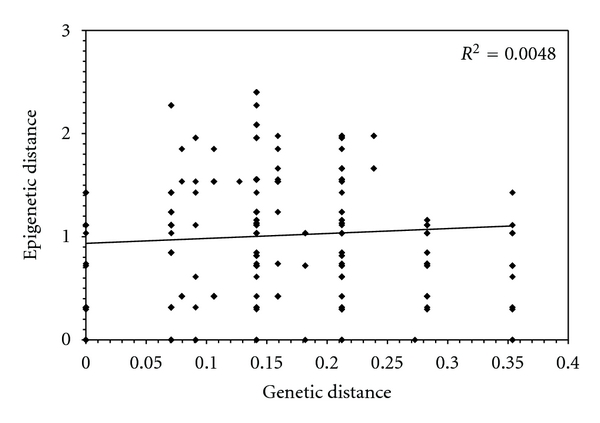
Relationship between the genotypes (genetic variation and microsatellite analysis) and the epigenotypes (methylation profiles difference and MSAP analysis).

Contrasting with genetic relationships among clones where variants are descendents of an ancestral genotype (Figure 2(b)), populations clustered in two distinct epigenetic groups separated by three epimutations (Figure 2(c)). No significant relationship was detected between genetic and epigenetic variation (Figure 3). For instance, individuals from two distinct lakes and harbouring the same genotype clustered in distinct epigenetic groups. Similarly, there is no relationship between genetic intrapopulation variability and epigenotypes. As an example, the six different genotypes from Barbotte Lake clustered into the same epigenetic group (Figure 2(c)). 

There is no indication that epigenetic profile is related to geographic position, hydrologic network (Figure 2(a)), or date of sampling (Table 1). Also, no difference in individual body size length (*P* = 0.26), body weight (*P* = 0.28), Fulton's K (*P* = 0.91), and GSI (*P* = 0.72) were detected among populations. In addition, the shared epigenetic profiles among populations are not correlated with the habitats characterization of lakes (Table 1). While there is no important temperature fluctuation among lakes, we observed a significant pH variation that is consistent with the two epigenetic groups (Table 1). This is a particularly important result, since it correlates the clustering of populations in two epigenetic groups to the variation of a local environmental condition. 

## 4. Discussion

The present study report an effect of the local environmental conditions on the variation of the methylation profile among genetically identical individuals belonging to different natural populations. This is a particularly important result, considering that most studies investigating the influence of the integration of the extrinsic signal of the environment on epigenetic variation were performed under control conditions (e.g., [14, 16, 17]) (but see [18]). This indicates that the variation of natural environmental conditions can lead to DNA methylation polymorphism at the population level. 

### 4.1. A Successful Generalist Lineage

The *C. eos-neogaeus* hybrid lineage studied here (lineage B6) is widespread in the south-western part of Quebec and is abundant in many populations from numerous watersheds [21]. The seven lakes under investigation are thought to be characterized by different environmental conditions of a variety of abiotic and biotic conditions (e.g., the oxygen concentration, the diets, the predation level, and the presence of competitors) [29]. Accordingly, each of the different lakes can be thought of as a different ecological niche. As a result, clonal lineage B6 can be characterized as a generalist lineage that is able to adjust in order to persist among many ecological niches. This situation has already been reported in northern Minnesota lakes (USA) and Algonquin Park lakes (Ontario) [23, 29]. Interestingly, *C. eos-neogaeus* hybrids from a single clonal lineage have been shown to present a high level of phenotypic variation [22]. Such variation of the phenotype in the absence of genetic variation has also been observed among *C. eos-neogaeus* hybrids from Quebec populations (B. Angers, unpublished data).

### 4.2. Environmentally Induced Epigenotypes

First, we did not detect any relationship between genotype and epigenotype. This is in accordance with a previous study that demonstrated pure (or facilitated) epigenetic variation in natural populations of *C. eos-neogaeus* hybrids [19]. More importantly, the genomic mutations detected are restricted to highly variable microsatellites loci, there is no mutation at mtDNA [21], and very few mutations were detected on AFLP loci [19]. This supports that the fragment variation detected with the MSAP analysis is due to difference in methylation not to DNA mutation.

Interestingly, the epigenetic polymorphism observed is shared among individuals of the same population in most instances. This suggests an influence of common environmental factors on the resulting epigenetic profiles or a long-term inheritance of epigenetic variation (modifications that could have been acquired before postglacial colonization). Considering the low probability of the inheritance of epigenetic variation across generations [33] and the absence of correlation between genetic and epigenetic polymorphism, the long-term heritability hypothesis can be ruled out. Accordingly, the observation of among lakes epigenetic variation suggests that current environmental conditions have an influence on the DNA methylation profiles among genetically identical individuals from different populations as opposed to hard-wired or germline dependent [34, 35]. In contrast with previous observations, the detection of the same epigenotype in different lakes indicates that the epigenetic polymorphisms observed are not the result of random variation [19]. More importantly, the correlation between the two epigenetic groups and the pH variation strongly support an effect of the local environmental conditions on the variation of methylation profile. Such pH variation may be caused by and/or will result in the variation of many other environmental factors potentially having respective or conjoint effects on the methylation polymorphism.

### 4.3. Revisiting the Importance of Heritability for Epigenetic Variation

Previous reports in the literature suggests that in order to be of importance in evolution, epigenetic changes must be heritable across generations [36–38]. In the situation for which an epimutation leading to a beneficial phenotypic modification appears in one generation and that the environmental conditions do not change in subsequent generations, the heritability of the new epigenetic mark may represent a transient step leading to genetic assimilation [39]. Although epimutations potentially represent a fast pathway toward adaptation [38], we do not believe that the main interest of epigenetic mechanisms is to mimic what is occurring at the adaptive genomic level. If heritable, both genetic and epigenetic polymorphisms are frozen. In temporally unstable or heterogeneous environments, such canalization of the phenotype does not seem beneficial [40]. Furthermore, heritability of epigenetic changes in vertebrates is not expected to be frequent considering the two phases of erasure prior to the initiation of zygote development [33]. Angers and coauthors [9] have recently identified some of the beneficial aspects of epigenetic mechanisms in that these processes may enable rapid and reversible changes in response to environmental perturbations. For instance, such is observed for the influence of the maternal behaviour on a glucocorticoid receptor gene promoter in the rat hippocampus [14]. Rather than passing on to the next-generation epimutations that may not be adaptive under new environmental regimes, selection might favour individuals with a plastic genome that easily adjusts epigenetically to environmental variables. Thus, the hard-wired genetic variation and the flexible epigenetic variation may be complementing each other by, respectively, leading to long-term and short-term adaptation.

## 5. Conclusion

While preliminary, these results appear to confirm that response of the genome when under variable environmental conditions leads to the formation of different epigenotypes. Each population presenting different epigenetic profiles can be seen as an acclimated epigenotype from a single flexible genetic lineage. It thus seems that this lineage has the potential to respond via epigenetic modifications such as DNA methylation when under variable environmental conditions. Even more importantly, this lineage potentially has the capacity to colonize different environments and/or the ability to adjust following a perturbation in the environment as expected from the long-term maintenance of multiple populations in this lineage. Thus, epigenetic processes may represent a molecular mechanism sustaining the GPG model.

## Figures and Tables

**Table 1:** Summary of ecological and molecular data. Lake environmental characteristics, individual morphometric characteristics, sampling size, genetic diversity (number of genotypes), and epigenetic diversity (number of epigenotypes).

Lakes	Geographic coordinates	Date of sampling	Habitat type*	Drainage	Altitude (m)	*T* (°C)	pH	Weight (g)	Length (cm)	K	GSI	Sampling size	Genotype	Epigenotype
Richer	45°50′35′′ N 74°11′39′′ W	2007-05-29	C	Nord	360	24	6.4	3.32 ± 0.94	6.93 ± 0.71	0.97 ± 0.05	7.33 ± 2.27	4	1	2
Merde	45°57′55.9′′ N 74°1′41,8′′ W	2007-05-28	B	L'Assomption	360	23	6.2	3.44 ± 1.36	6.78 ± 0.77	1.04 ± 0.07	11.37 ± 2.7	4	3	4
Barbotte	46°5′36′′ N 73°52′7′′ W	2007-05-30	C	L'Assomption	280	22	6.5	2.16 ± 0.4	6.04 ± 0.41	0.97 ± 0.05	7.19 ± 2.5	8	6	2
Jonction	45°46′37′′ N 74°34′29′′ W	2007-06-01	C	Rouge	340	24	7.1	2.38 ± 0.56	6.08 ± 0.54	1.05 ± 0.07	9.74 ± 0.71	4	1	1
Dépotoir	45°50′41.6′′ N 74°33′20.9′′ W	2007-05-31	B	Rouge	320	25	7.1	1.85 ± 0.38	5.65 ± 0.45	1.01 ± 0.03	10.73 ± 0.53	2	1	1
Saumons	45°59′38′′ N 74°18′21′′ W	2007-06-16	A	Nord	490	22	7.0	3.2 ± 0.26	6.8 ± 0	1.01 ± 0.08	6.51 ± 1.31	2	1	2
Saad	45°54′51.4′′ N 74°1′41.3′′ W	2007-06-16	D	L'Assomption	320	24	6.8	1.89 ± 0.18	5.9 ± 0.2	0.92 ± 0.008	4.48 ± 2.54	2	1	1

*Habitats characterization according to Schlosser et al. [29]: A: pond of moderate depth, B: a shallow beaver pond, C: a moderately deep area of open water upstream from a beaver dam, and D: pond of moderate depth with flooded standing and fallen tree.
